# Effect of Anemia in Postoperative Outcomes of Autologous Breast Reconstruction Surgery

**DOI:** 10.29252/wjps.8.3.285

**Published:** 2019-09

**Authors:** Hossein Masoomi, Matthew R. Greives, Andrew D. Cantor, Erik S. Marques

**Affiliations:** 1 Division of Plastic and Reconstructive Surgery, Department of Surgery, McGovern Medical School at the University of Texas, Health Science Center at Houston, Houston, USA;; 2 Division of Pediatric Plastic Surgery, Department of Pediatric Surgery, McGovern Medical School at the University of Texas Health Science Center at Houston, Houston, USA

**Keywords:** Anemia, Postoperative, Autologous, Breast, Reconstruction

## Abstract

**BACKGROUND:**

The true effects of anemia on postoperative surgical outcomes in autologous breast reconstruction surgery are unknown. We intended to evaluate the effect of chronic anemia on surgical outcomes in autologous breast reconstruction surgeries using a large national database.

**METHODS:**

Using the Nationwide Inpatient Sample database, we examined the clinical data of patients who underwent immediate or delayed autologous breast reconstruction surgery from 2012 to 2014. Univariate and multivariate regression analyses were performed to independently evaluate the effect of chronic anemia on postoperative outcomes.

**RESULTS:**

Totally, 55,839 patients underwent autologous breast reconstruction surgery (immediate: 40% vs. delayed: 60%) during this period. Overall, 6.0% of patients had chronic anemia at the time of surgery. Compared with patients without chronic anemia, patients with chronic anemia had a significantly higher complication rate (19.8% vs. 9.4%) and a longer mean length of hospital stay (5.4 vs. 3.7 days). Postoperative complications were significantly higher in patients with chronic anemia compared with patients without chronic anemia except for venous thromboembolism (VTE) and fat necrosis. Multivariate regression analyses demonstrated that chronic anemia was independently associated with an increased overall complication rate (adjusted odds ratio: 2.20). Also, multivariate regression analyses showed that chronic anemia was an independent risk factor of all the evaluated postoperative complications except VTE, stroke and fat necrosis.

**CONCLUSION:**

This study demonstrated that chronic anemia was a significant predictor factor of morbidity in autologous breast reconstruction including flap failure. Correction of anemia prior to breast reconstruction may help reduce poor surgical outcomes related to chronic anemia.

## INTRODUCTION

Preoperative anemia, even mild anemia, has been shown as an independent factor associated with an increased risk of morbidity and mortality in patients undergoing major non-cardiac and cardiac surgery.^1^^,^^2^ Preoperative anemia is usually regarded as a risk factor because of its association with increased perioperative transfusions of blood components.^3^^,^^4^ Breast cancer patients are at risk of anemia and interestingly, decreased hemoglobin (Hb) levels are reportedly correlated with poor treatment outcomes and survival in patients of breast cancer.^5^^-^^7^

The causes of anemia in cancer patients are often multifactorial, including chemotherapy, radiation-induced myelosuppression, bleeding, bone marrow infiltration by cancer invasion, nutritional deficiencies, and cytokine-mediated anemia.^5^ There are limited studies evaluating the effect of preoperative anemia on postoperative outcomes in autologous breast reconstruction with various reports in postoperative outcomes.^8^^-^^10^ Obtaining a better understanding of the effect of anemia on surgical outcomes may stimulate plastic surgeons to optimize hemoglobin levels prior to elective autologous breast reconstruction surgery. We intended to evaluate the effect of preoperative anemia independently in immediate surgical outcomes in detail in autologous breast reconstruction surgeries using the Nationwide Inpatient Sample (NIS) database.

## MATERIALS AND METHODS

The NIS is the largest publicly available all-payer inpatient health care database in the United States, yielding national estimates of hospital inpatient stays. The NIS is part of a family of databases and software tools developed for the Healthcare Cost and Utilization Project (HCUP). Unweighted, it contains information over 7 million hospital stays each year across the country. Weighted, it estimates more than 35 million hospitalizations nationally. The NIS contains clinical and resource use information included in a typical discharge abstract, with safeguards to protect the privacy of individual patients, physicians, and hospitals as required by data sources. The NIS is comprised of a nationally represented sample of approximately 20% of US community hospitals excluding rehabilitation and long-term acute care hospitals. 

The NIS is drawn from all States participating in HCUP, representing more than 97 percent of the US population. The NIS can be weighted to produce national estimates. Data elements within the NIS allow determination of all procedures performed during a given hospital admission. It also contains discharge information on inpatient hospital stay, including patient characteristics, length of stay, specific in-hospital postoperative complications, total hospital charges and observed in-hospital mortality. The NIS includes charge information for all patients, regardless of payer, including persons covered by Medicare, Medicaid, private insurance, and the uninsured. The NIS database has no information available on complications occurring after discharge.^11^

Using the NIS database, we analyzed discharge data on patients who underwent autologous breast reconstruction surgery from 2012 to 2014. We used International Classification of Disease ninth revision, Clinical Modification (ICD-9-CM) procedure codes^12^ of autologous breast reconstructions including latissimus dorsi myocutaneous flap (85.71), pedicle transverse rectus abdominis myocutaneous flap (85.72), free transverse rectus abdominis myocutaneous flap (85.73), free deep inferior epigastric artery perforator flap (85.74), free superficial inferior epigastric artery flap (85.75), free gluteal artery perforator flap (85.76) and others including non-otherwise specified (85.71 and 85.79) to identify our patient population. 

Also, to identify immediate versus delayed autologous breast reconstruction, patients who had ICD-9-CM procedure codes of mastectomies (85.42, 85.43, 85.44, 85.46, 85.46, 85.47 and 85.48) in combination with autologous breast reconstruction codes were included in the immediate breast reconstruction group. Preoperative factors that were analyzed including patient’s characteristics, patient’s comorbidities, reconstruction-type, reconstruction timing, prior chemotherapy, prior radiotherapy and teaching status of hospital. 

In this database, patients with anemia at admission were identified using ICD-9-DM diagnosis codes of 280.1-281.9 (iron deficiency anemia), 285.21-285.29 (anemia of chronic disease) and 285.9 (unspecified including essential, normocytic anemia not due to blood loss). We evaluated the frequency and effect of chronic anemia in postoperative complications in this patient population. Also, we compared the surgical outcomes of patients with and without chronic anemia. Postoperative complications were identified using ICD-9-DM diagnosis codes.

All statistical analyses for the NIS database were conducted using SAS (version 9.4, SAS institute, Cary, North Carolina). As the NIS database is a stratified sample of 20% of all the US hospitals, discharge weight (DISCWT) was used to create national estimates for all analyses. Univariate and multivariate regression analyses were performed to identify independently the effect of chronic anemia in postoperative complications following autologous breast reconstructive surgery patients. Step-wise forward logistic regressions were conducted for each postoperative outcome adjusting for patient’s characteristics (age and race), comorbidities, history of prior radiation or chemotherapy, type of breast reconstruction (free versus pedicled flap) and timing of reconstruction (immediate versus delayed). Statistical significance was set at *p* values <0.05. Mean values are presented as mean**±**standard deviation (SD).

## RESULTS

During 2012-2014, a total of 55,840 patients underwent autologous breast reconstruction in the United States. Examining patient characteristics, the mean age was 52 years old and 11.3% of the patients were over 65 years old (Table 1), while the majority of the patients were Caucasian (71.0%). All the evaluated comorbidities were significantly higher in anemic cohort group except history of smoking which there was not significant between groups. Overall, most common comorbidity was hypertension (26.9).

**Table 1:** Patients’ characteristics who underwent autologous breast reconstruction in the United States from 2012 to 2014

**Characteristics**	**Overall**	**Without Anemia**	**With Anemia **	***p*** ** value **
Number	55,840	49,520	6,320	
Age (year)				
Mean	52±10	52±10.1	51.7±10.6	0.46
Median	52			
Mode	52			
Over 65 (%)	11.3	11.4	10.6	0.17
Race				
White	71.0	71.7	59.2	<0.01
Black	13.5	13.0	21.8	
Hispanic	8.2	8.2	9.6	
Asian\Islander	3.1	3.1	4.6	
Native American	0.3	0.3	0.3	
Other	3.9	3.8	4.4	
Comorbidity				
Diabetes mellitus	7.8	7.5	13.3	<0.01
Hypertension	26.9	26.3	37.1	<0.01
Congestive heart failure	0.6	0.5	2.0	<0.01
Chronic lung disease	8.9	8.6	13.2	<0.01
Chronic kidney disease	0.7	0.6	2.8	<0.01
Liver disease	0.6	0.6	1.1	<0.01
Smoker	17.9	17.9	17.5	0.58
Obesity	10.0	9.6	15.5	<0.01
Prior chemotherapy	11.7	11.4	16.1	<0.01
Prior radiation	16.7	16.7	16.9	0.81
Teaching hospitals	80.0	80.2	78.5	0.08
Immediate reconstruction	40.0	39.9	42.3	<0.01
Free Flap Reconstruction	53.4	52.8	62.5	<0.01

The majority of the operations were performed at teaching hospitals (80%). Considering the timing of breast reconstruction, the majority of patients underwent delayed breast reconstruction (60%). The two most common types of autologous breast reconstruction were DIEP flap (31.6%) and Latissimus dorsi myocutaneous flap (31%). The overall frequency of chronic anemia was 6.0%. The highest frequency of patients with chronic anemia was found in the cohort undergoing free TRAM flap reconstruction (8.3%, Table 2). 

**Table 2:** Frequency of autologous breast reconstruction type and anemia rate

**Autologous breast reconstruction types **	**Overall** **(%)**	**Anemia Rate ** **(%)**	**With Anemia ** **(%)**	**Without Anemia ** **(%)**
LD^a^ flap	31.0	4.3	31.6	22.7
Pedicled TRAM^b^ flap	11.6	6.6	11.5	13.2
Free TRAM flap	16.0	8.3	15.5	22.7
DIEP^c^ flap	31.6	6.7	31.4	36.0
SIEA^d^ flap	0.7	6.5	0.7	0.8
GAP^e^ flap	0.6	2.8	0.7	0.3
Not otherwise specified	8.4	3.0	8.7	4.3

a) LD: Latissimus dorsi myocutaneous flap, b) TRAM: Pedicle transverse rectus abdominis myocutaneous flap; c) DIEP: Free deep inferior epigastric artery perforator flap; d) SIEA: Free superficial inferior epigastric artery flap; e) GAP: Free gluteal artery perforator flap

When compared to patients without chronic anemia, patients with chronic anemia had a higher overall complication rate (19.79% vs. 9.35%; *p*<0.01), longer mean length of stay (5.4 days vs. 3.8 days; *p*<0.01), higher mean total hospital charges ($101,967 vs. $78,712; *p*<0.01) and in-hospital mortality rate (0.15% vs. 0.05%, *p*=0.01). All the evaluated complications were significantly higher in chronic anemia patients except venous thromboembolism (VTE) and fat necrosis which was not significant between groups (Table 3).

**Table 3:** Comparison of outcomes in autologous breast reconstruction with and without chronic anemia

**Outcomes**	**Without chronic anemia ** **%**	**With chronic anemia ** **%**	***p*** ** value**
Overall complication	9.35	19.79	<0.01
Urinary tract infection	0.87	1.69	<0.01
Pneumonia	0.33	0.77	<0.01
Acute respiratory failure	0.52	0.92	<0.01
Acute kidney injury	0.55	2.45	<0.01
Venous thromboembolism	0.10	0.15	0.41
Myocardial infarction	0.03	0.15	<0.01
Stroke	0.05	0.15	0.01
Wound complication	1.19	3.83	<0.01
Hematoma	2.48	4.91	<0.01
Fat necrosis	0.89	0.61	0.10
Seroma	1.14	1.84	<0.01
Flap failure	1.09	2.15	<0.01
Blood transfusion	5.76	27.15	<0.01
Length of hospital stay (days)	3.8	5.4	<0.01
Mean total hospital charges ($)	78,712	101,967	<0.01
In-hospital mortality	0.05	0.15	0.01

Table 4 and Table 5 show the univariate and multivariate regression analyses evaluating the effect of chronic anemia in autologous breast reconstruction. Univariate regression analyses showed that all the evaluated postoperative complications were significantly higher in anemic group compared with non-anemic group except VTE and fat necrosis which were similar in both groups (Table 4). Using stepwise logistic regression analyses (Table 5) showed that chronic anemia was an independent risk factor for increased overall complication rate (adjusted odds ratio [AOR]: 2.20). 

**Table 4:** Univariate regression analysis for the outcomes in patients who had chronic anemia following autologous breast reconstructive surgery

**Outcomes **	**AOR (95% CI)** ^a^	***p*** ** value**
Urinary tract infection	1.95 (1.47–2.58)	<0.01
Pneumonia	2.31 (1.52–3.52)	<0.01
Acute respiratory failure	1.77 (1.21–2.58)	<0.01
Acute kidney injury	4.53 (3.53–5.83)	<0.01
Venous thromboembolism	1.47 (0.59–3.67)	0.41
Myocardial infarction	5.38 (1.96–14.82)	<0.01
Stroke	3.23 (1.24–8.44)	0.02
Wound complication	3.32 (2.73–4.03)	<0.01
Hematoma	2.03 (1.72–2.40)	<0.01
Seroma	1.62 (1.24–2.12)	<0.01
Fat necrosis	0.68 (0.44–1.07)	0.10
Flap failure	1.96 (1.55–2.55)	<0.01
Blood transfusion	6.12 (5.52–6.66)	<0.01
Overall complication	2.39 (2.18–2.62)	<0.01

a) AOR, Adjusted odds ratio; CI: Confidence interval

**Table 5:** Multivariate regression analyses^a^ for the outcomes in patients who had preoperative anemia following autologous breast reconstructive surgery

**Outcomes **	**AOR (95% CI)** ^b^	***p*** ** value**
Urinary tract infection	1.59 (1.19–2.11)	<0.01
Pneumonia	1.85 (1.20–2.86)	<0.01
Acute respiratory failure	1.64 (1.10–2.44)	0.02
Acute kidney injury	3.01 (2.31–3.96)	<0.01
Venous thromboembolism	1.06 (0.41–2.70)	0.90
Myocardial infarction	4.25 (1.53–11.82)	<0.01
Stroke	1.54 (0.54–4.37)	0.42
Wound complication	3.08 (2.51–3.80)	<0.01
Hematoma	1.73 (1.44–2.08)	<0.01
Seroma	1.69 (1.28–2.22)	<0.01
Fat necrosis	0.72 (0.46–1.14)	0.16
Flap failure	1.90 (1.47–2.45)	<0.01
Blood transfusion	5.21 (4.76–5.70)	<0.01
Overall complication	2.20 (2.00–2.42)	<0.01

a) Stepwise logistic regression analyses considering patient’s characteristics (age and race), patient’s comorbidities, chemotherapy and radiation therapy status, free versus pedicled breast reconstruction, delayed versus immediate reconstruction; b) AOR, Adjusted odds ratio; CI: Confidence interval; NS: Not significant.

Similarly, multivariate regression analysis showed that chronic anemia was an independent risk factor for the majority of postoperative complications including blood transfusion (adjusted odds ratio [AOR]: 5.21), MI (AOR: 4.25), wound complications (AOR: 3.08), acute kidney disease (AOR: 3.01), flap failure (AOR: 1.90), pneumonia (AOR: 1.85), hematoma (AOR: 1.73), seroma (AOR: 1.69), acute respiratory failure (AOR: 1.64), and urinary tract infection (AOR: 1.59). However, chronic anemia was not a predictor risk factor of VTE, stroke and fat necrosis.

## DISCUSSION

This represents the largest retrospective study evaluating the effect of chronic anemia in autologous breast reconstructive surgery. To our knowledge, this is the largest study demonstrating the independent adverse effect of preoperative anemia in immediate postoperative outcomes in autologous breast reconstruction. This study demonstrated that patients with a history of chronic anemia had a 2.1 times higher complication rate, 1.4 times longer mean length of stay and 1.3 times higher mean total hospital charges compared to patients without chronic anemia. Therefore, correction of anemia in this patient population, where possible, may improve perioperative outcomes.

In the current study, using the NIS database, the overall rate of chronic anemia was 6.0%, which was lower compared with 16-18% anemia rate in previously published studies evaluating preoperative anemia in breast reconstructions.^8^^-^^10^ However, the anemia rate in this patient population was within the range of general surgical population. Shanker *et al.*,^13^ in a systemic review of the literature evaluating the prevalence and outcomes of anemia in surgery, reported the prevalence of preoperative anemia from 5% in geriatric women with hip fracture to 58 % in colorectal cancer patients. 

Hb level varies depending to the stage of breast cancer treatment. Lee *et al.*,^5^ in a study evaluating the effect of Hb level with survival outcomes in patients with breast cancer, showed that Hb level was the highest at the beginning of the 1-year period following treatment and then gradually decreased to 12.0 mg/dL at the end of the 1-year period. After adjusting patient characteristics, comorbidities, type of breast reconstruction and timing of reconstruction, stepwise forward logistic analyses showed that chronic anemia was a significant risk factor for all evaluated postoperative complications including flap failure except VTE, stroke and fat necrosis (Table 4). 

In a similar study, Nelson *et al.*^9^ in a single institution study evaluated the impact of anemia on microsurgical breast reconstruction and showed that a higher incidence of medical complications in cohorts with Hb<10; however, preoperative anemia was not associated with increased risk of flap related complication. One of the main contributing factors related with increased postoperative complications (e.g. acute kidney injury, respiratory complications, and wound complications) could be the known effect of anemia in decreasing oxygen-carrying capacity, which may contribute to both flap and patient morbidity.^14^

In another study, Sarhane *et al.*^10^ analyzed the effect of anemia on postoperative outcomes in only immediate breast reconstruction (both implant-based and autologous reconstruction) and demonstrated an independent association between preoperative anemia and 30-day morbidity (AOR: 1.38, 95% CI, 1.02-1.85; *p*<0.01). There have been multiple studies evaluating the effect of anemia in free flap survival with controversial findings. Studies have discussed the benefits of normovolemic hemodilution in flaps based on the theory that decreased viscosity increases cardiac output and thus arterial flow.^15^^,^^16^

Additionally, animal models have demonstrated that normovolemic anemia may be even protective to free ﬂaps regarding to increased ischemic time.^17^^,^^18^ Also, there have been studies which showed that preoperative anemia was not associated with free ﬂap failure in breast and general reconstruction.^9^^,^^19^ However, the current study showed that chronic anemia was a predictor risk factor of flap failure (AOR: 1.90). Similar to our study, Hill *et al.*,^20^ in evaluation of 156 free flap in microvascular reconstruction found that preoperative anemia was a significant predictor of flap failure and vascular thrombosis. Also, they mentioned that the risk of flap failure in patients with preoperative anemia was likely primarily related to vascular thrombosis. 

Preoperative anemia is also known as a risk factor due to its association with increased perioperative transfusions of blood components.^3^^,^^4^ Our study showed that preoperative anemia was associated with a significant increase risk of blood transfusion (AOR: 5.21 [anemic patient: 27.10% vs. non-anemic: 5.76%,] *p*<0.01). Consistent with our study, Nelson *et al.*,^8^ showed patients with preoperative anemia had a three times higher chance of blood transfusion (18%) compared with non-anemic patients (6%, *p*<0.0001). 

Our study also showed that patients with chronic anemia had a significantly longer hospital stay and total hospital charges compared with non-anemic patients. These findings were consistent with other studies evaluating the effect of anemia in breast reconstruction surgeries.^9^^,^^10^ This might be as consequence of lower functional capacity for recovery and rehabilitation^4^^,^^8^ as well as the mentioned higher rate of complications in these patients with anemia. 

The limitations of this study are similar to other retrospective studies using a large database. The NIS database is an inpatient database without outpatient follow up; therefore, we were unable to capture readmissions and post-discharge complications. In addition, as the ICD-9 codes were used to define the anemia preoperatively; therefore, we were unable to identify the severity of anemia (Hb level) which might influence the outcomes. However, there have been studies in breast reconstruction and non-breast reconstruction showing the increasing postoperative complications with more declining in Hb level.^1^^,^^9^

Also, we were unable to evaluate the effect of anemia in functional recovery in this patient population. Nelson *et al.*,^8^ in a prospective study of 179 patients who underwent autologous breast reconstruction, showed (i) preoperatively, anemic patients had a significantly worse physical, mental and overall health compared with non-anemic patients and (ii) preoperative anemia adversely impacted the recovery of breast reconstruction patients. Lastly, as breast reconstruction is an elective procedure, it is unclear how many patients presented for breast reconstruction and were denied due to anemia or anemia was corrected prior to surgery. 

Despite the mentioned limitations, to our knowledge, this is the largest study concentrating on the effect of anemia in immediate surgical outcomes in autologous breast reconstruction using a nationwide database. This study demonstrated that chronic anemia was a significant predictor factor of morbidity in autologous breast reconstruction including flap failure as well as increased length of hospital stay and increased hospital charges. Identifying anemic patients and correction of anemia prior to breast reconstruction may help to reduce poor surgical outcomes related to chronic anemia. Future prospective studies would be required to evaluate the effect of anemia in autologous breast reconstruction in detail with consideration of the limitations in this study.

## CONFLICT OF INTEREST

The authors declare no conflict of interest.
